# Supplementary figures and images for: Hepatitis B virus RNAs co-opt ELAVL1 for stabilization and CRM1-dependent nuclear export
Source: PLoS Pathog. 2024 Feb 2;20(2):e1011999. doi: 10.1371/journal.ppat.1011999 (PMC10866535; doi:10.1371/journal.ppat.1011999)

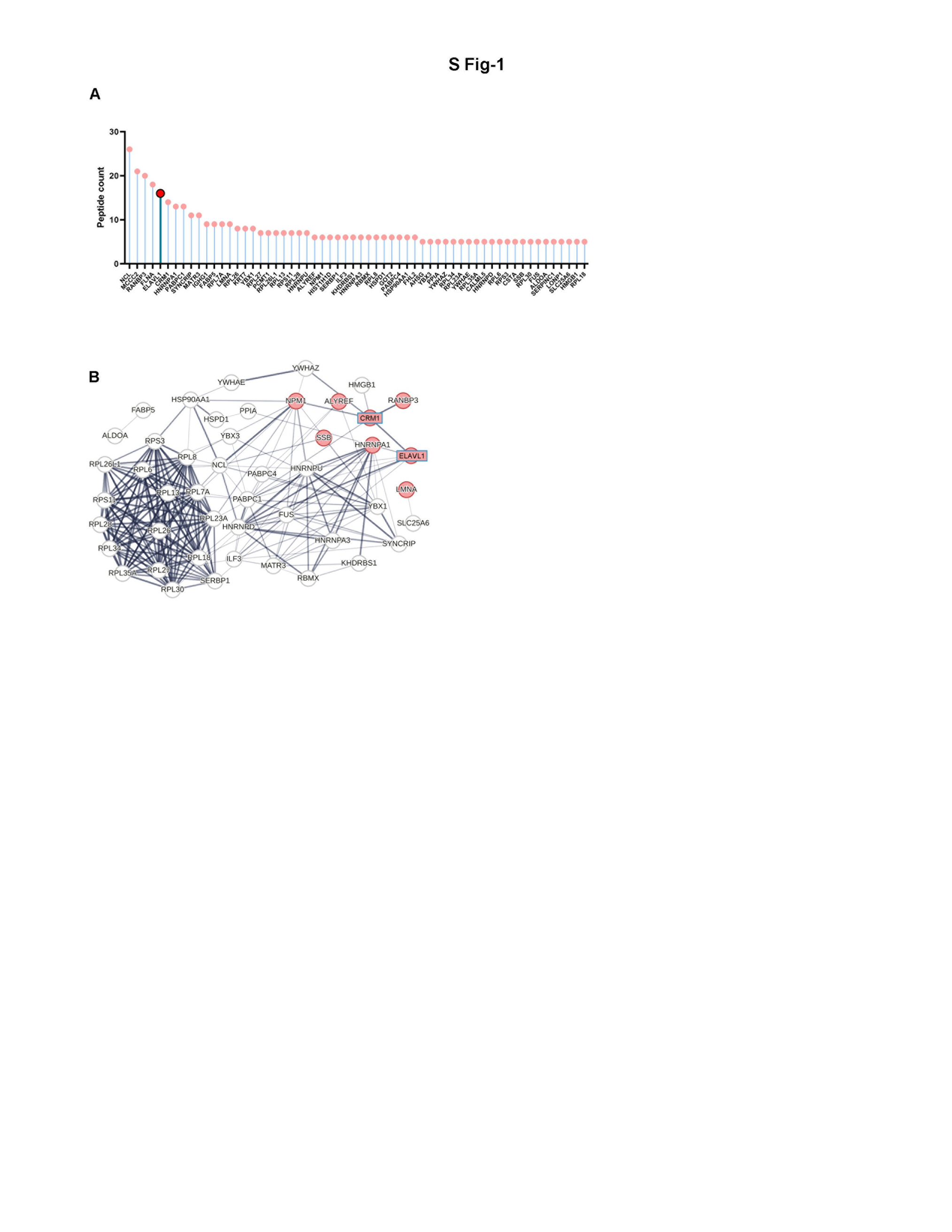

Supplement: S1 Fig — (A) Peptide count of the pgRNA binders identified by the RNA-pulldown-LC-MS/MS. (B) Gene ontology analysis (cluster analysis) for the pgRNA binders’ network. Red node indicates the cluster of nucleocytoplasmic transport regulators. (TIF) [file ppat.1011999.s001.tif]

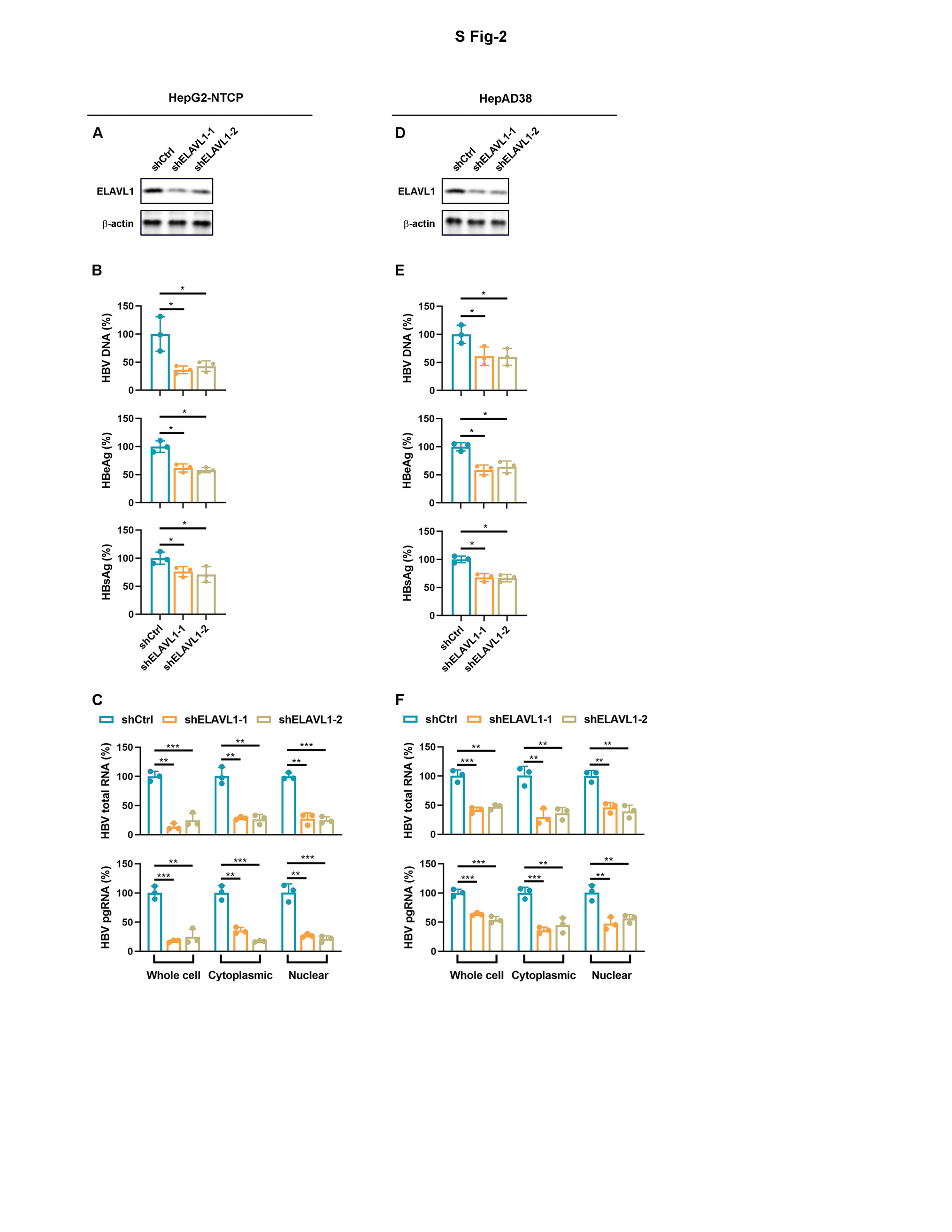

Supplement: S2 Fig — (A-C) ELAVL1 knockdown HepG2-NTCP cells were pretreated with 2.5% DMSO for 2 days following HBV infection at an MOI of 200 and were maintained with DMEM containing 2.5% DMSO for 7 days. (D-F) ELAVL1 knockdown HepAD38 cells were maintained in DMEM medium containing 2% DMSO for 2 days. (A and D) Knockdown efficiency was confirmed by WB. (B and E) The levels of HBV DNA in culture supernatant were determined by qPCR (% of shCtrl). The levels of HBeAg and HBsAg in culture supernatant were determined by ELISA (% of shCtrl). (C and F) Subcellular levels of HBV RNAs in cytoplasm and nucleus were determined by qPCR (% of shCtrl). Graphs show mean ± SD. *p < 0.05; **p < 0.01; ***p < 0.001. (TIF) [file ppat.1011999.s002.tif]

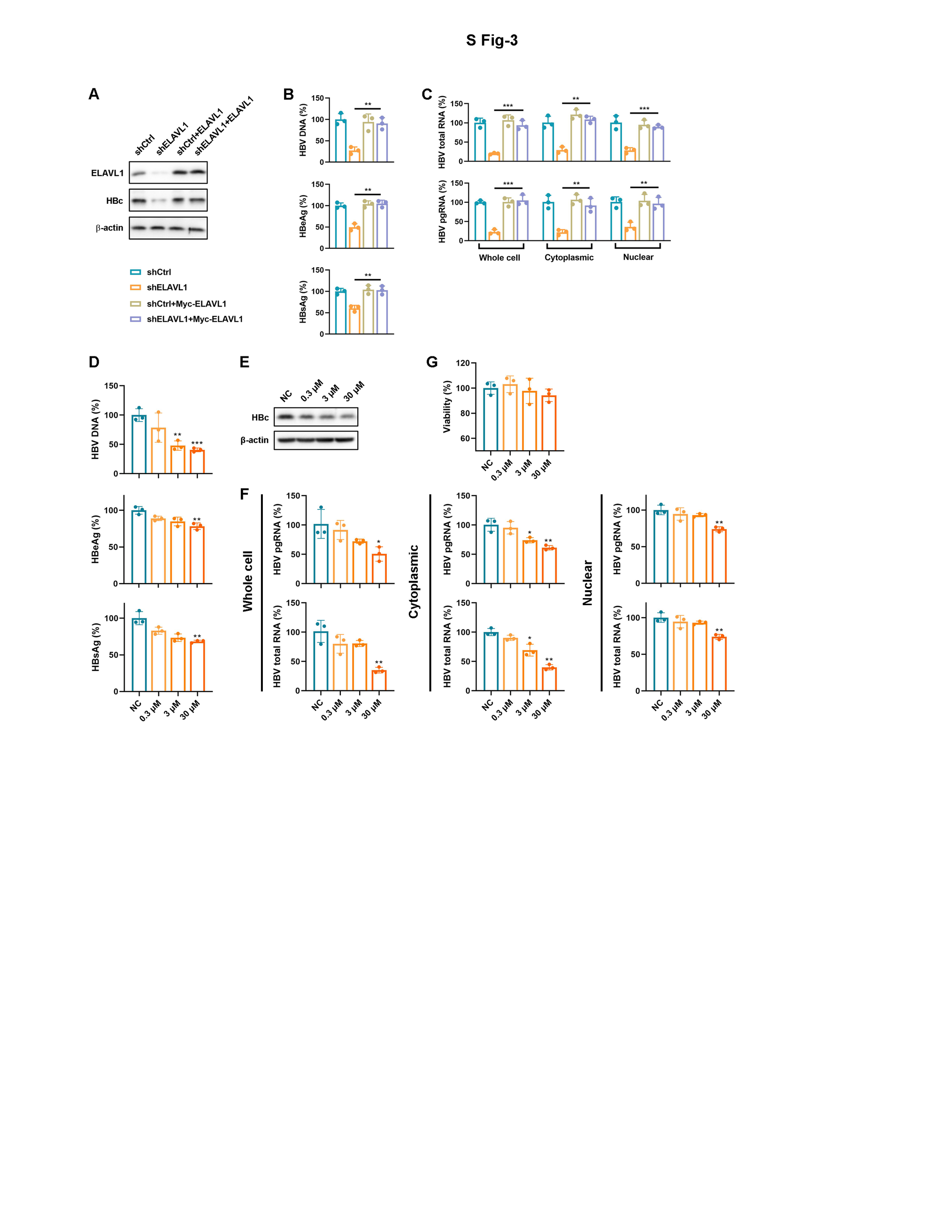

Supplement: S3 Fig — (A-C) The lentivirus expressing ELAVL1 infected HepG2-NTCP cells with stable ELAVL1 knockdown. The cells were pretreated with 2.5% DMSO for 2 days following HBV infection at an MOI of 200 and were maintained with DMEM containing 2.5% DMSO for 7 days. (A) Levels of ELAVL1 and HBc were evaluated by WB. (B) The levels of HBV DNA in culture supernatant were determined by qPCR (% of shCtrl). The levels of HBeAg and HBsAg in culture supernatant were determined by ELISA (% of shCtrl). (C) Subcellular levels of HBV RNAs in cytoplasm and nucleus were determined by qPCR (% of shCtrl). (D-G) HepAD38 cells were treated with CMLD-2 as indicated dose for 48 hours. (D) Levels of HBV-DNA in supernatants were determined by qPCR (% of NC). Levels of secreted HBeAg and HBsAg were determined by ELISA (% of NC). (E) Levels of HBc were determined by WB. (F) Subcellular levels of HBV RNAs in cytoplasm and nucleus were determined by qPCR (% of NC). (G) Cell viability was evaluated by CCK-8 assay. Graphs show mean ± SD. *p < 0.05; **p < 0.01; ***p < 0.001. (TIF) [file ppat.1011999.s003.tif]

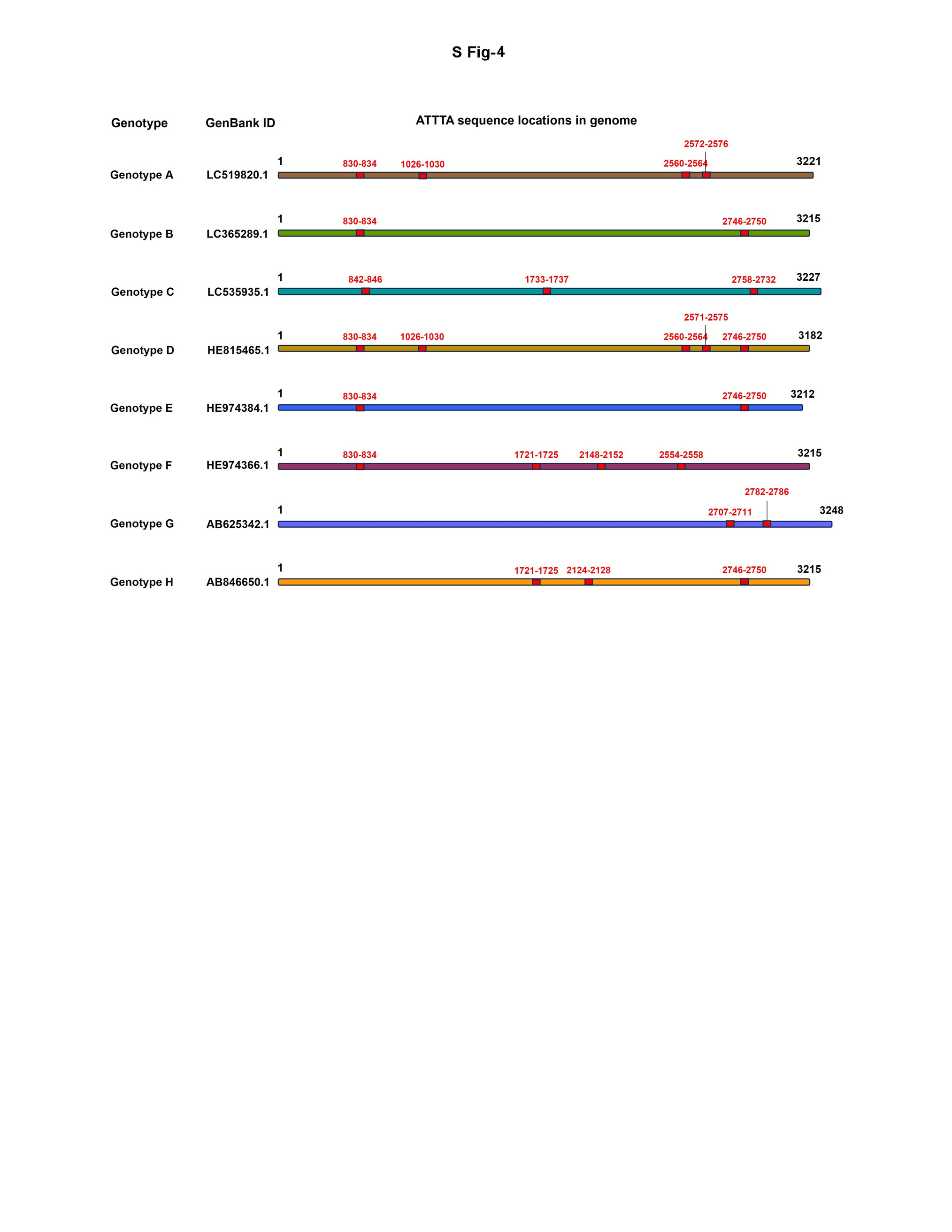

Supplement: S4 Fig — (TIF) [file ppat.1011999.s004.tif]

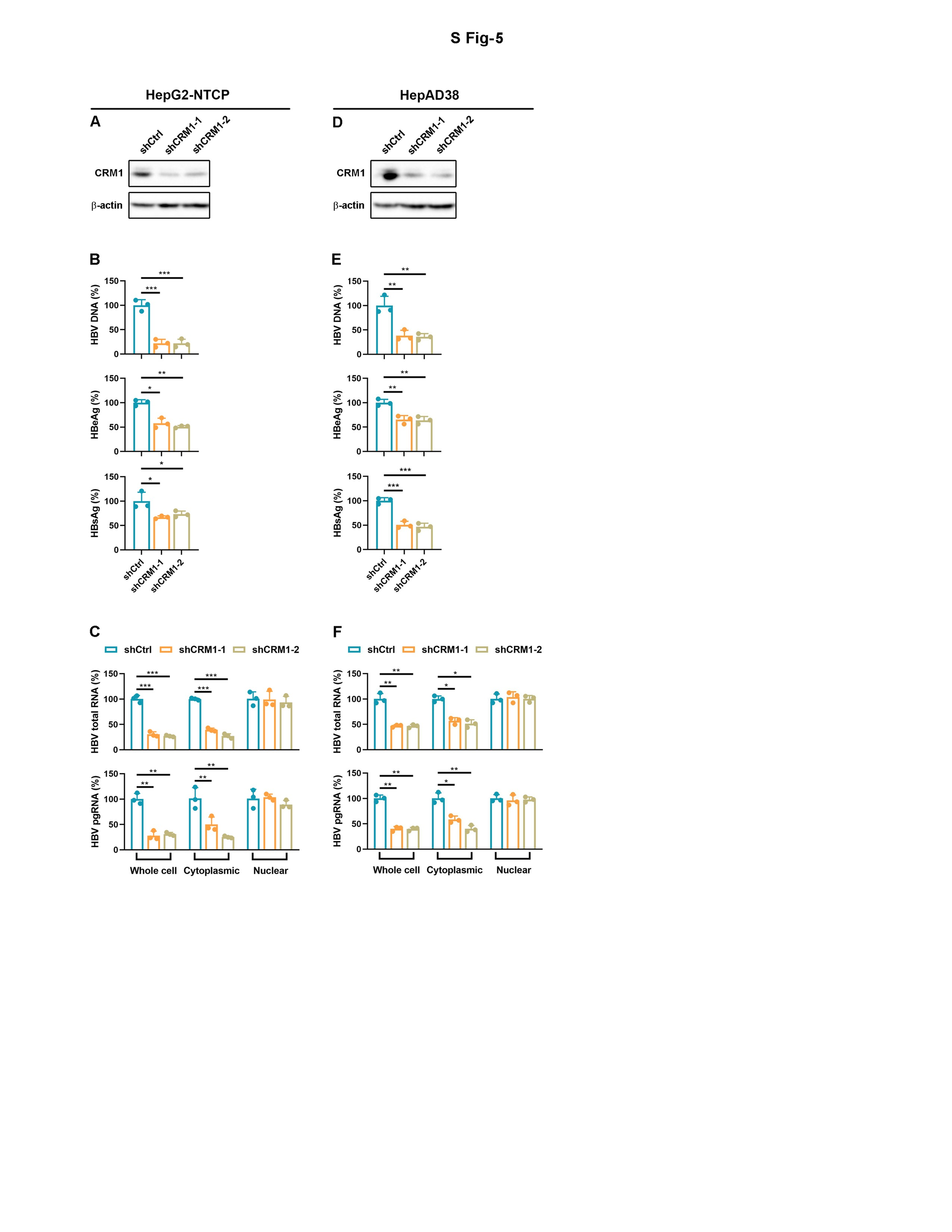

Supplement: S5 Fig — (A-C) CRM1 knockdown HepG2-NTCP cells were pretreated with 2.5% DMSO for 2 days following HBV infection at an MOI of 200 and were maintained with DMEM containing 2.5% DMSO for 7 days. (D-F) CRM1 knockdown HepAD38 cells were maintained in DMEM medium containing 2% DMSO for 2 days. (A and D) Knockdown efficiency were confirmed by WB. (B and E) The levels of HBV DNA in culture supernatant were determined by qPCR (% of shCtrl). The levels of HBeAg and HBsAg in culture supernatant were determined by ELISA (% of shCtrl). (C and F) Subcellular levels of HBV RNAs in cytoplasm and nucleus were determined by qPCR (% of shCtrl). Graphs were shown as mean ± SD. *, p < 0.05; **, p < 0.01; ***, p < 0.001. (TIF) [file ppat.1011999.s005.tif]

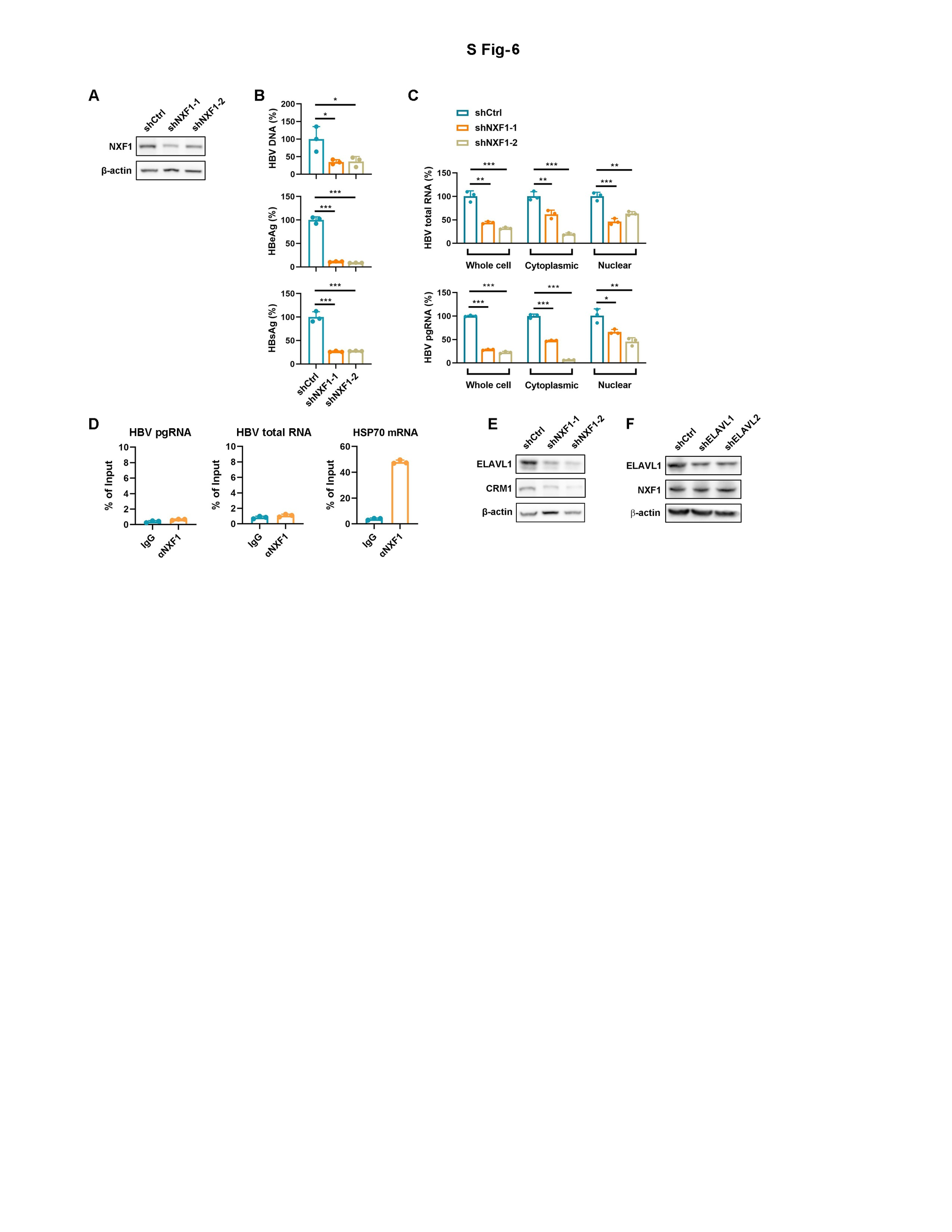

Supplement: S6 Fig — (A-E) NXF1 knockdown HepG2-NTCP cells were pretreated with 2.5% DMSO for 2 days following HBV infection at an MOI of 200 and were maintained with DMEM containing 2.5% DMSO for 7 days. (A) Knockdown efficiency of was confirmed by WB. (B) Levels of HBV DNA in supernatants were determined by qPCR (% of shCtrl). Secreted HBeAg and HBsAg levels were determined by ELISA (% of shCtrl). (C) Subcellular levels of HBV RNAs in cytoplasm and nucleus were determined by qPCR (% of shCtrl). Graphs show mean ± SD. *p < 0.05; **p < 0.01; ***p < 0.001. (D) The binding of NXF1-HBV RNA, and NXF-HSP70 were detected by RIP assay. (E) Levels of ELAVL1, and CRM1 were determined by WB. (F) ELAVL1 knockdown HepG2-NTCP cells were pretreated with 2.5% DMSO for 2 days following HBV infection at an MOI of 200 and were maintained with DMEM containing 2.5% DMSO for 7 days. Levels of NXF1 were evaluated by WB. (TIF) [file ppat.1011999.s006.tif]

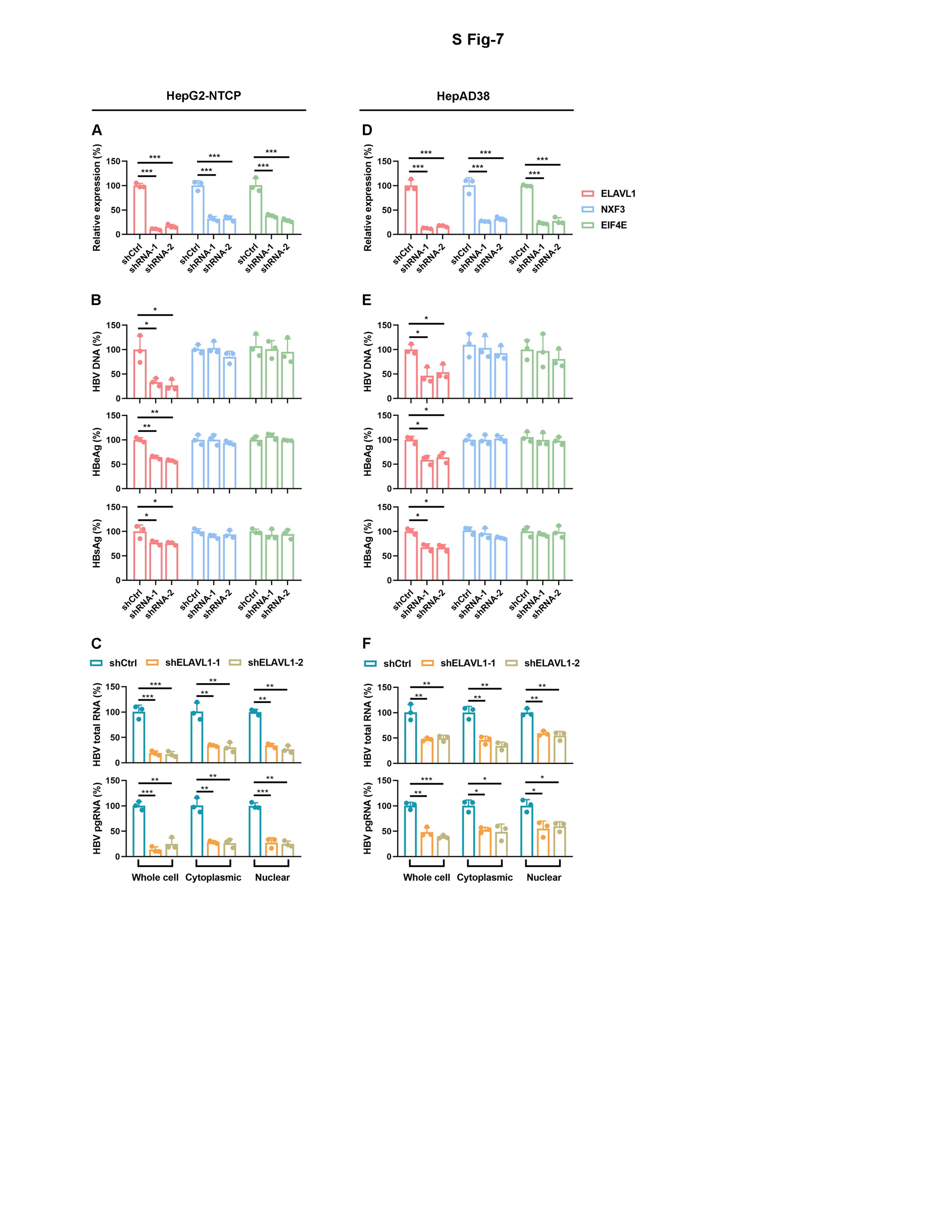

Supplement: S7 Fig — (A-C) The HepG2-NTCP-K7 cells were infected with lentivirus expressing ELAVL1, NXF3, or EIF4E targeted shRNA. The cells were infected with HBV at an MOI = 200 and were harvested at 7 days post HBV infection. (B-D) The HepAD38 cells were infected with lentivirus expressing ELAVL1, NXF3, or EIF4E targeted shRNA. The cells were harvest at 2 days post infection. (A and D) Knockdown efficiency was confirmed by qPCR (% of shCtrl). (B and E) The levels of HBV DNA in culture supernatant were determined by qPCR. The levels of HBeAg and HBsAg in culture supernatant were determined by ELISA (% of shCtrl). (C and F) The subcellular distribution of HBV RNAs was determined by qPCR (% of shCtrl). Graphs show mean ± SD. *p < 0.05; **p < 0.01; ***p < 0.001. (TIF) [file ppat.1011999.s007.tif]

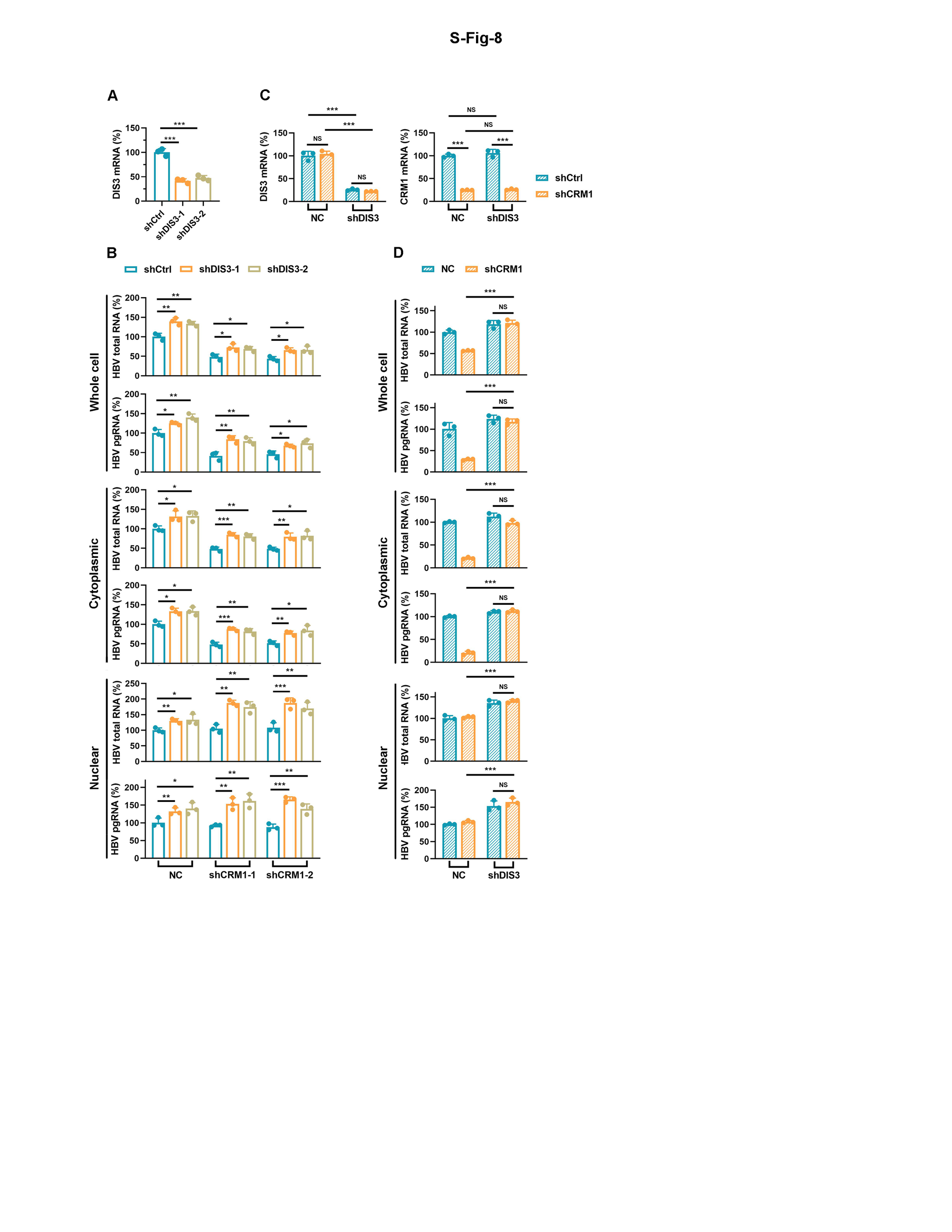

Supplement: S8 Fig — (A and B) CRM1 knockdown HepG2-NTCP cells were transfected with the DIS3 targeted shRNAs and were infected with HBV at an MOI = 200. The cells were maintained in DMEM medium containing with 2.5% DMSO for 7 days. (A) Knockdown efficiency of the DIS3 targeted shRNAs was confirmed by qPCR (% of shCtrl). (B) Subcellular levels of HBV RNAs in cytoplasm and nucleus were determined by qPCR (% of shCtrl). (C and D) DIS3 knockdown HepG2-NTCP cells were transfected with the CRM1 targeted shRNAs and were infected with HBV at an MOI = 200. The cells were maintained in DMEM medium containing with 2.5% DMSO for 7 days. (C) Levels of DIS3 and CRM1 mRNA were determined by qPCR (% of shCtrl). (D) Subcellular levels of HBV RNAs in cytoplasm and nucleus were determined by qPCR (% of shCtrl). Graphs were shown as mean ± SD. *, p <0.05; **, p < 0.01; ***, p < 0.001. (TIF) [file ppat.1011999.s008.tif]
