# Supplementary material for: Hepatitis B virus RNAs co-opt ELAVL1 for stabilization and CRM1-dependent nuclear export
Source: PLoS Pathog. 2024 Feb 2;20(2):e1011999. doi: 10.1371/journal.ppat.1011999 (PMC10866535; doi:10.1371/journal.ppat.1011999)
Supplement: S1 Table — (PDF) [file ppat.1011999.s009.pdf]

S1 Table. Critical commercial kits

Critical commercial kits	Source	Identifier
Ultrapure RNA Kit	CWBIO	Cat #: CW0581
ReverTra Ace qPCR RT Master Mix with gDNA Remover	TOYOBO	Cat #: FSQ-301
FastSmart Essential DNA Green Master Mix	ROCH	Cat #: 06924204001
Nuclear and Cytoplasmic Extraction Reagents Kit	Beyotime	Cat #: P00028
RNA Purification Kit	SIMGEN	Cat #: 5401050
T7 mScript Standard mRNA Production System	CELLSCRIPT	Cat #: C-MSC11610
RNA 3' End Desthiobiotinylation Kit	Thermo Scientific	Cat #: 20163
RNAScope Sample Preparation Kit	Advanced Cell Diagnostics	Cat #: 322381
RNAscope Multiplex Fluorescent Detection Kit	Advanced Cell Diagnostics	Cat #: 323110
RNAscope Probe V-HBV-GTD	Advanced Cell Diagnostics	Cat #: 441351
HBV DNA Diagnostic Kit	Sansure	
ELISA HBV Test Kit (HBeAg)	KHB	
ELISA HBV Test Kit (HBsAg)	KHB	
Dual-Luciferase Reporter Assay System	Promega	Cat #: E2940
