# Supplementary material for: Hepatitis B virus RNAs co-opt ELAVL1 for stabilization and CRM1-dependent nuclear export
Source: PLoS Pathog. 2024 Feb 2;20(2):e1011999. doi: 10.1371/journal.ppat.1011999 (PMC10866535; doi:10.1371/journal.ppat.1011999)
Supplement: S2 Table — (PDF) [file ppat.1011999.s010.pdf]

S2 Table. Antibodies

Antibodies	Source	Identifier
Rabbit anti-HBc	Self-made	N/A
Rabbit anti-HBs	NOVUS Biology	Cat #: NB10062652
Rabbit anti-ELAVL1	proteintech	Cat#:11910-1-AP
Rabbit anti-CRM1	ABclonal	Cat #: A19625
Rabbit anti-DIS3	Proteintech	Cat#:14689-1-AP
Rabbit anti-NXF1	Proteintech	Cat#:10328-1-AP
Rabbit anti-ANP32A	ABclonal	Cat#: A5768
Rabbit anti-ANP33B	Huaan Biotechnology	Cat#: ET109-89
Rabbit anti-β-actin	ABclonal	Cat #: AC026
Mouse anti-Myc	BIOPRIMACY	Cat #: PMK112M
Mouse anti-His	BIOPRIMACY	Cat #: PMK0115
Anti-rabbit IgG, HRP-linked	CellSignaling Technology	Cat #: 7074
Anti-mouse IgG, HRP-linked	CellSignaling Technology	Cat #: 7076
Rabbit IgG	Proteintech	Cat #: B900610
Mouse IgG	Proteintech	Cat #: B900620
