# Supplementary material for: Hepatitis B virus RNAs co-opt ELAVL1 for stabilization and CRM1-dependent nuclear export
Source: PLoS Pathog. 2024 Feb 2;20(2):e1011999. doi: 10.1371/journal.ppat.1011999 (PMC10866535; doi:10.1371/journal.ppat.1011999)
Supplement: S3 Table — (PDF) [file ppat.1011999.s011.pdf]

S3 Table. Oligonucleotides

Oligonucleotides	Sequence
**siELAVL1-1**	5'-TACCAGTTTCAATGGTCATAA-3'
**siELAVL1-2**	5'-TTGTTAGTGTACAACTCATTT-3'
**siCRM1-1**	5'-GCTCAAGAAGTACTGACACAT-3'
**siCRM1-2**	5'-CCATTGTAAAGCGACTTCAAA-3'
**siANP32A-1**	5'-CCTATTGTGATTTGACTGTTT-3'
**siANP32A-2**	5'-CCTGAAGATGAGGGAGAAGAT-3'
**siANP32B-1**	5'-GCTTACCTACTTGGATGGCTA-3'
**siANP32B-2**	5'-CCACCCAAAGAGCCAAAGAAT-3'
**siRRP4**	5'-GAGAAGCTCATTGCATCTGTT-3'
**siEXOSC4**	5'-CGCTCCCAGATTGATATCTAT-3'
**siDIS3**	5'-CCCGAACTCATAGATCGTCTT-3'
**siRRP6**	5'-GCATGTCCTTTCCAACTGGAA-3'
**shELAVL1-1 forward primer**	5'-CCGGTACCAGTTTCAATGGTCATAACTCGAGT TATGACCATTGAAACTGGTATTTTTG -3'
**shELAVL1-1 reverse primer**	5'-AATTCAAAAATACCAGTTTCAATGGTCATAAC TCGAGTTATGACCATTGAAACTGGTA -3'
**shELAVL1-2**	5'- CCGGTTGTTAGTGTACAACTCATTTCTCGAGAA

**forward primer**	ATGAGTTGTACACTAACAATTTTTG -3’
**shELAVL1-2 reverse primer**	5’- AATTCAAAAATTGTTAGTGTACAACTCATTTCT CGAGAAATGAGTTGTACACTAACAA -3’
**sh*Elavl1* forward primer**	5’-ACCCGAGGTTGAATCTGCAAAGCTCTCGAGAG CTTTGCAGATTCAACCTCG-3’
**sh*Elavl1* reverse primer**	5’-AACCGAGGTTGAATCTGCAAAGCTCTCGAGAG CTTTGCAGATTCAACCTCG-3’
**shCRM1-1 forward primer**	5’-CCGGGCTCAAGAAGTACTGACACATCTCGAGA TGTCAGTCAGTACTTCTTGAGCTTTTTG-3’
**shCRM1-1 reverse primer**	5’-AATTCAAAAAGCTCAAGAAGIACIGACACATC TCGAGATGTGTCAGATCTTCTTGAGCT-3’
**shCRM1-2 forward primer**	5’-CCGGCCATTGTAAAGCGACTTCAAACTCGAGT TTGAAGTCGCTTTACAATGGTTTTTG-3’
**shCRM1-2 reverse primer**	5’-AATTCAAAAACCATTGTAAAGCGACTTCAAAC TCGAGTTTGAAGTCGCTTTACAATGG-3’
**shDIS3-1 forward primer**	5’-CCGGCCCTGCCATCAGGAATGTAATCTCGAGAT TACATTCCTGATGGCAGGGTTTTTG-3’
**shDIS3-1 reverse primer**	5’-AATTCAAAAACCCTGCCATCAGGAATGTAATC TCGAGATTACATTCCTGATGGCAGGG-3’

**shDIS3-2 forward primer**	5’-CCGGCCCGAACTCATAGATCGTCTTCTCGAGA AGACGATCTATGAGTTCGGGTTTTTG-3’
**shDIS3-2 reverse primer**	5’-AATTCAAAAACCCGAACTCATAGATCGTCTTC TCGAGAAGACGATCTATGAGTTCGGG-3’
**shNXF3-1 forward primer**	5’-CCGGCCAGAGCAACTTTATGTGGTACTCGAGT ACCACATAAAGTTGCTCTGGTTTTTG-3’
**shNXF3-1 reverse primer**	5’-AATTCAAAAACCAGAGCAACTTTATGTGGTAC TCGAGTACCACATAAAGTTGCTCTGG-3’
**shNXF3-2 forward primer**	5’-CCGGACAAGCTCTTTGTGCGGGATACTCGAGT ATCCCGCACAAAGAGCTTGTTTTTTG-3’
**shNXF3-2 reverse primer**	5’-AATTCAAAAAACAAGCTCTTTGTGCGGGATAC TCGAGTATCCCGCACAAAGAGCTTGT-3’
**shNXF1-1 forward primer**	5’-CCGGCGCGAACGATTTCCCAAGTTACTCGAGT AACTTGGGAAATCGTTCGCGTTTTTG-3’
**shNXF1-1 reverse primer**	5’-AATTCAAAAACGCGAACGATTTCCCAAGTTAC TCGAGTAACTTGGGAAATCGTTCGCG-3’
**shNXF1-2 forward primer**	5’-CCGGTCAGAAGATTGAGCCTCACTGCTCGAGC AGTGAGGCTCAATCTTCTGATTTTTG-3’
**shNXF1-2 reverse**	5’-AATTCAAAAATCAGAAGATTGAGCCTCACTGC

**primer**	TCGAGCAGTGAGGCTCAATCTTCTGA-3’
**shEIF4E-1 forward primer**	5’-CCGGCGGCTGATCTCCAAGTTTGATCTCGAGA TCAAACTTGGAGATCAGCCGTTTTTG-3’
**shEIF4E-1 reverse primer**	5’-AATTCAAAAACGGCTGATCTCCAAGTTTGATC TCGAGATCAAACTTGGAGATCAGCCG-3’
**shEIF4E-2 forward primer**	5’-CCGGCCACTCTGTAATAGTTCAGTACTCGAGTA CTGAACTATTACAGAGTGGTTTTTG-3’
**shEIF4E-2 reverse primer**	5’-AATTCAAAAACCACTCTGTAATAGTTCAGTAC TCGAGTACTGAACTATTACAGAGTGG-3’
**pgRNA forward primer**	5’-CTGGGTGGGTGTTAATTTGG-3’
**pgRNA reverse primer**	5’-TAAGCTGGAGGAGTGCGAAT-3’
**Total RNA forward primer**	5’-CCGTCTGTGCCTTCTCATCTGC-3’
**Total RNA reverse primer**	5’-ACCAATTTATGCCTACAGCCTCC-3’
**CRM1 forward primer**	5’-CTCCCTAATCAAGTGTGGGATAG-3’
**CRM1 reverse**	5’-TGTTTCAGGATCTTTCAGTATATCCA-3’

**primer**	
**ELAVL1 forward primer**	5’-CAGGCGCAGAGATTCAGG-3’
**ELAVL1 reverse primer**	5’-GGTTGTAGATGAAAATGCACCA
**RRP4 forward primer**	5’-TGGCTCGCAAGCCTCTTAG-3’
**RRP4 reverse primer**	5’-TGTGTCCGTAGTGATTGTATCCC-3’
**EXOSC4 forward primer**	5’-CTCTTGTCGGACCAGGGCTA-3’
**EXOSC4 reverse primer**	5’-TGCCCTGCTCAATGTAGGC-3’
**DIS3 forward primer**	5’-AGTCAGTGGGTAGCACCATCT-3’
**DIS3 reverse primer**	5’-AGCATTCGTTCTGTCTCTTCTTC-3’
**RRP6 forward primer**	5’-CGGATGATCAGCAACCGC-3’
**RRP6 reverse primer**	5’-TAGCATTTTCTAGCGCGGC-3’
**NXF3 forward**	5’-AGTGTGCACGACCTTCTCG-3’

**primer**	
**NXF3 reverse primer**	5’-TGGGGAACAATTCCAGGAT-3’
**EIF4E forward primer**	5’-TTTTGGGCTCTGTACAACCA-3’
**EIF4E reverse primer**	5’-CCCACATAGGCTCAATACCATC-3’
**HSP70 forward primer**	5’-AGGACATCAGCCAGAACAAG-3’
**HSP70 reverse primer**	5’-GTAGAAGTCGATGCCCTCAAA-3’
**β-actin forward primer**	5’-ATCGTGCGTGACATTAAGGAG-3’
**β-actin reverse primer**	5’-GGAAGGAAGGCTGGAAGAGT-3’
**human snU2 forward primer**	5’-ATGGATTTTTGGAGCAGGGAGA-3’
**human snU2 reverse primer**	5’-GCACCGTTCCTGGAGGTACTG-3’
**human snU6 forward primer**	5’-CTCGCTTCGGCAGCACA-3’
**human snU6**	5’-AACGCTTCACGAATTTGCGT-3’

**reverse primer**	
**mouse snU1 forward primer**	5’-GGAGATACCATGATCACGAAGG-3’
**mouse snU1 reverse primer**	5’-AGTCGAGTTTCCCGCATTT-3’
