# Supplementary material for: Hepatitis B virus RNAs co-opt ELAVL1 for stabilization and CRM1-dependent nuclear export
Source: PLoS Pathog. 2024 Feb 2;20(2):e1011999. doi: 10.1371/journal.ppat.1011999 (PMC10866535; doi:10.1371/journal.ppat.1011999)
Supplement: S4 Table — (PDF) [file ppat.1011999.s012.pdf]

S4 Table. Chemicals

Chemicals	Source	Identifier
Leptomycin B	APEXBIO	Cat #: B6907
CMLD-2	MedChemExpress	Cat #: HY-124828
PEI MAX 40K	Polysciences	Cat #: 24765-1
Lipofectamine RNAiMAX	Invitrogen	Cat #: 13778075
