# Supplementary material for: Hepatitis B virus RNAs co-opt ELAVL1 for stabilization and CRM1-dependent nuclear export
Source: PLoS Pathog. 2024 Feb 2;20(2):e1011999. doi: 10.1371/journal.ppat.1011999 (PMC10866535; doi:10.1371/journal.ppat.1011999)
Supplement: Attachment — Submitted filename: Response-2023-12-9.docx [file ppat.1011999.s013.docx]

Dear editors,

We thank the reviewers and editors for their helpful comments, and are pleased that the reviewers regarded our work as “an exhaustive study”, “well-designed”, “well-executed” and “a thorough investigation”. In this revision, we provided additional data involving quantification of intrahepatic replicative DNA in Elavl1 knockdown AAV-HBV mice, effects of ELAVL1 in HBV infected primary human hepatocytes, a rescue experiment using ELAVL1 overexpression in the background of ELAVL1 CRISPR KO cells, and revised our manuscript based on constructive suggestions from the reviewers. Please find out point-by-point response below in *italic blue*.

**Part I - Summary**

Please use this section to discuss strengths/weaknesses of study, novelty/significance, general execution and scholarship.

Reviewer #1: This is an exhaustive study to establish ELAVL1 and its interacting network as regulators of HBV RNA stability and nuclear export. The authors first identified host proteins associated with HBV pgRNA, followed by pathway analysis to focus on ELAVL1 protein for experimental testing. Knocking down ELAVL1 expression from HBV infected HepG2/NTCP cells reduced extracellular HBV DNA, HBeAg and HBsAg, and intracellular core protein and replicative DNA. That was associated with reduced 3.5-kb HBV RNAs and total HBV RNAs in both nuclear and cytoplasmic fractions. Such reductions in HBV markers can be rescued by reintroducing exogenous ELAVL1 (Figs. S3A-C). Similar effect of ELAVL1 silencing was observed in HepAD38 cell line of stable HBV transfection (less dramatic) and in C57B/6 mice transduced with AAV-1.2mer HBV genome. Further study revealed binding of ELAVL1 to 3.5-kb HBV RNAs via AU-rich elements (AREs). Since CRM1 is a known nuclear export receptor for ELAVL1 and its bound RNAs, and considering a previous publication implicating CRM1 in nuclear export of encapsidated pgRNA, the authors examined and confirmed a role of CRM1 in regulating the cytoplasmic (but not nuclear) abundance of HBV RNAs (the use of leptomycin B and a resistant CRM1 mutant made the story more convincing). Moreover they found a role of protein ligands ANP32A and ANP32B in recruiting CRM1 to the HBV RNA-ELAVL1 complex. In the absence of ELAVL1 nuclear HBV RNAs were found to be degraded by DIS3 and RRP6 RNases. While a previous report suggested a role of NXF1 in pgRNA nuclear export, the authors found that silencing NXF1 also reduced ELAVL1 protein level, thus raising the possibility that NXF1 shRNA worked through ELAVL1 instead. Overall this is a solid work centering on ELAVL1 but with ramifications about upstream and downstream events. But since mutating all the four AREs in pgRNA only reduced pgRNA by about 50% (Fig. 4J), ELAVL1 does not explain everything. That is especially true for subgenomic RNAs (no ARE in 0.7-kb RNA and only one ARE in 2.4- and 2.1-kb RNAs). Thus a role of PRE (post-transcriptional regulatory element) in promoting nuclear export of HBV RNAs, especially the subgenomic ones, probably remains valid.

Reviewer #2: ELAVL1 and its effects on the abundance. Further mechanistic studies led to the model that ELAVL1 interacte with ANP32A, ANP32B which tethers to CRM1 for nuclear export and also protects exome mediated RNA decay. Coherent as the model may look like, the authors' interpretation of the effects of ELAVL1 on HBV life cycle might be over-simplified.

Reviewer #3: The authors have done a thorough investigation in the discovery and characterization of ELAVL1 as a host dependency factor that impacts HBV biology. In particular, they used an unbiased proteomics approach that identified ELAVL1 binding to HBV pregenomicRNA (pgRNA) and through a series of experiments they showed that this host factor affects export of HBV RNAs from the nucleus to the cytoplasm by recruiting the nuclear export receptor CRM1 and by protecting HBV RNAs from degradation. Overall, this study is well-designed and the experiments are well-executed supporting the conclusions. Based on the above comments I would recommend this article for publication after the authors address some points mentioned below.

**Part II – Major Issues: Key Experiments Required for Acceptance**

Please use this section to detail the key new experiments or modifications of existing experiments that should be absolutely required to validate study conclusions.

Generally, there should be no more than 3 such required experiments or major modifications for a "Major Revision" recommendation. If more than 3 experiments are necessary to validate the study conclusions, then you are encouraged to recommend "Reject".

Reviewer #1: In vivo studies are less artificial and important. For Fig. 3, why was there no data on serum HBV DNA and/or intracellular replicative DNA?

*Thanks for pointing this issue out. We determined the levels of intrahepatic replicative DNA as suggested by the reviewer. HBc associated DNA in Elavl1 knockdown AAV-HBV mice was evaluated. The data indicated a reduction in intrahepatic HBc-associated DNA, suggesting that Elavl1 knockdown suppressed HBV replication in vivo. This data is shown in new Fig 3C.*

***Elavl1 knockdown suppressed intrahepatic HBc-associated DNA in vivo.*** *The C57B/6 mice were transduced with AAV-1.2 HBV along with AAV expressing Elavl1-targeted shRNA. The mice were sacrificed 28 days post transduction. Intrahepatic HBc-associated HBV DNA were assessed using qPCR.*

Reviewer #2: As the AU-rich element is wide spread in the transcriptome, the effects of ELAVL1 perturbation may be widespread and there may be indirect effects on other host genes that cause the observed manifestation in various levels of HBV life cycle. Indeed, as a recent review pointed out (PMC7555251), ELAVL1 was shown to regulate many cell-cycle related proteins and knock down of ELAVL1 (PMC9316910) led to reduced proliferation in HepAD38 cells. Did the authors looked into this issue?

*This is really an important issue. In our cell culture experiments, we aimed to circumvent the influence of ELAVL1 on the cell cycle. To achieve this, we consistently applied DMSO treatment in the cell culture medium to induce cell cycle arrest. As we demonstrated in the manuscript, prior to HBV infection, ~95% HepG2-NTCP cells were seeded in petri dishes and treated with 2.5% DMSO for 2 days. The HBV infected HepG2-NTCP cells were treated with 2.5% DMSO persistently for 5 days or 7 days as indicated in figure legends. Thus, any effects of proliferation reduction resulting from ELAVL1 knockdown on HepAD38 and HepG2-NTCP could not affect our investigations. We added the corresponding discussion in our revised manuscript (Line 289-294).*

*To further confirm this, we conducted a CCK-8 assay to evaluated the impact of ELAVL1 knockdown on the viability of HepAD38 and HepG2-NTCP infected with HBV. The data revealed that ELAVL1 knockdown had no effect on the viability of HepAD38 and HepG2-NTCP infected with HBV.*

***ELAVL1 knockdown does not affect the viability of HepAD38 and HepG2-NTCP cells infected with HBV which were treated with DMSO persistently to prevent cell proliferation.*** *(A) HepAD38 cells infected with lentivirus expressing ELAVL1-targeted shRNA were seeded into 96-well plates and treated with 2% DMSO persistently for 2 days. (B) HepG2-NTCP cells infected with lentivirus expressing ELAVL1 targeted shRNA were seeded into 96-well plate and subsequently infected with HBV at an MOI of 200 after being treated with 2.5% DMSO for 2 days. The cells were then maintained in DMEM medium supplemented with 2.5% DMSO for 7 days. Cell viability was evaluated using a CCK-8 assay.*

There are several additional issues that needs clarification.

1. Have the authors attempted to stain pgRNA using RNAscope together with ELAVL1 to validate the RNA-protein interaction?

*Thank you for the advice. We tried the co-staining of pgRNA and ELAVL1, but could not obtain good results. While RNAscope excels in visualizing RNA or DNA, simultaneously validating RNA-protein interactions poses a significant challenge due to methodological constraints. As proteinase was used to treat the samples, we could not get a strong signal of ELAVL1 staining. Since RNA pulldown, RIP, REMSA and mutagenesis assays were performed, we believe that the interaction between pgRNA and ELAVL1 is valid.*

2. In Figure 3F, the pgRNA positive rate in the AAV-1.2HBV model is 100% which is astonishing. Have the authors done additional controls to validate its specificity?

*The positive rate of the HBV RNA, as depicted in Figure 3F, was initially normalized to shCtrl. However, in order to prevent potential misinterpretation, we have revised the normalization method to absolute values and updated the data in new Fig 3G.*

*As shown in the representative images blow, the overall HBV RNA positive rate is about 95%, which is consistent with HBsAg staining.*

3. In most of the RNAscope FISH results, the signal is not puntate. Is it because of the limited resolution of the microscope or too much signal amplification was performed? What fluorescent microscope was used? As the RNAscope assay has many different versions, please specify the catalog number and the technical detail such as the fluorescent channel used.

*As the RNAscope images presented above, the detected signals exhibit a high degree of abundance and intensity, appearing as punctate structures in the raw images captured at a high resolution.*

*The detailed information of RNAscope kits has been provided in Supplementary Table 1. (RNAScope Sample Preparation Kit (Advanced Cell Diagnostics, Cat #: 322381); RNAscope Multiplex Fluorescent Detection Kit (Advanced Cell Diagnostics, Cat #: 323110); RNAscope Probe V-HBV-GTD (Advanced Cell Diagnostics, Cat #: 441351)).*

*The confocal laser scanning microscope we used to detect signals in cell was NIKON Eclipse Ti (software: Eclipse C2). The fluorescent channels we used were DAPI (EX 405 nm, EM 417-477 nm); probe dye 570 (EX 561 nm, EM 570-690 nm).*

*The confocal laser scanning microscope we used to detect signals in tissue was Leica TCS SP8 (software: LAS X Version 3.4.2.18368). The fluorescent channels we used were DAPI (EX 405 nm, EM 417-480 nm); probe dye 570 (EX 587 nm, EM 570-690 nm).*

*We have incorporated this information into the revised manuscript.*

4. The authors claims that ELAVL1 mediates a post-transcriptional regulation on HBV RNA. Although this assertion of plausible, necessary work is still needed to rule out the possibility of transcriptional regulation. Actually, this is not impossible considering the multi-faceted influence of ELAVL1 knockdown.

*Thank you very much for your suggestion. Initially, we observed a significant decrease in RNA stability upon knockdown of ELAVL1 using Actinomycin D chase experiments (Fig 2F). To further confirm if ELAVL1 play a role in transcriptional regulation, we investigated the effects of ELAVL1 knockdown on the HBV enhancer/promotor activity. The results revealed that ELAVL1 knockdown had no effect on the activity of HBV enhancer/promotor. The data were shown in new Fig 2.*

*The effects of ELAVL1 knockdown on the HBV enhancer/promotor activity.* *Huh7 cells were transfected with ELAVL1 siRNAs 24 hours prior to transfection of the HBV promoter luciferase reporter plasmids pGL-3-S1-promoter, pGL-3-S2-promoter, pGL-3-enhancer I/X- promoter, and pGL-3-enhancer II/precore/core- promoter respectively. Firefly luciferase activity (renilla luciferase activity as reference) was measured 48 hours after transfection (% of siCtrl).*

5. The effects of CMLD-2 on viral antigen and pgRNA seem to be weak, especially considering the Ki50 of this compound is 350nM.

*Thanks for pointing this issue out. In relation to CMLD-2 treatment, our primary emphasis is not to showcase it as a robust candidate for anti-HBV therapy. Rather, our objective is to provide a proof of concept illustrating that targeting ELAVL1 holds promise as a potential approach to suppress HBV. We added this statement in the revised manuscript (Line 51-52, 354-357)*

Reviewer #3: In Figure 1 the authors used a moderate and transient siRNA approach and the study would have benefited from a clean ELAVL1 CRISPR KO followed by a rescue experiment using ELAVL1 overexpression in the background of the KO cells. They already have the overexpression construct as shown in supplementary figure 3.

*The suggestion of employing a clean ELAVL1 CRISPR knockout followed by a rescue experiment involving ELAVL1 overexpression in the background of the knockout cells is quite insightful. We constructed ELAVL1 knockout HepG2-NTCP cell line and performed a rescue experiment using ELAVL1 overexpression. However, the attempt to utilize CRISPR for achieving a single clone have faced challenges, probably due to the crucial role of ELAVL1 in maintaining cellular functions.*

*Nevertheless, we conducted the experiment by using pooled cells. As demonstrated below, ELAVL1 knockout lead to a suppression of HBV secreted antigens production and HBV replication. Subsequent investigations demonstrated a reduction in both nuclear and cytoplastic HBV RNA levels all of which were effectively rescued by ELAVL1 overexpression.*

*ELAVL1 supports HBV replication. (A-C) The ELAVL1 expressing lentivirus infected HepG2-NTCP cells with ELAVL1 knockout. The cells were pretreated with 2.5% DMSO for 2 days following HBV infection at an MOI of 200 and were maintained with DMEM containing 2.5% DMSO for 5 days. (A) Levels of ELAVL1 were evaluated by WB. (B)* *The levels of HBV DNA in culture supernatant were determined by qPCR (% of shCtrl). The levels of HBeAg and HBsAg in culture supernatant were determined by ELISA (% of shCtrl). (C) Subcellular levels of HBV RNAs in cytoplasm and nucleus were determined by qPCR (% of shCtrl).*

CMLD-2 as an ELAVL1 inhibitor has been reported to have anti-tumor activity in multiple cancer cell lines. All the studies here use cancer cell lines (HepG2) and the study would be more relevant if inhibition of ELAVL1 (either genetic or pharmacological) could be validated in primary human hepatocytes. In addition, a more thorough cytotoxicity assay and not only b-actin in WB would be useful and easy to generate in cell lines. Especially, when 30uM of a compound is used in Fig 3D and the reduction of HBc is not as robust.

*Thank you for the suggestion. We knocked down ELAVL1 by siRNA in primary human hepatocytes. In align with the investigations in HepG2-NTCP cells, the data indicated that ELAVL1 knockdown reduced levels of secreted antigens, HBV proliferation, and HBV RNA. The data were incorporated into new Fig 1.*

***ELAVL1 knockdown suppressed HBV replication in primary human hepatocytes.*** *The primary human hepatocytes were transfected with ELAVL1 targeted siRNA 2 days before HBV infection (MOI = 200). Samples were collected 5days post HBV infection. (A) Knockdown efficiency was evaluated by qPCR (% of siCtrl). (B) The levels of HBV DNA in culture supernatant were determined by qPCR (% of siCtrl). The levels of HBeAg and HBsAg in culture supernatant were determined by ELISA (% of siCtrl). (C) The levels of intracellular HBV RNA were assessed by qPCR.*

*Cytotoxicity assay was performed and presented in new Fig. 2F as shown below:*

**Part III – Minor Issues: Editorial and Data Presentation Modifications**

Please use this section for editorial suggestions as well as relatively minor modifications of existing data that would enhance clarity.

Reviewer #1: 1. The initial screening for host factors interacting with HBV RNAs (Fig. S1 and Fig. 1) was based on pgRNA, which and pcRNA has 3 more AREs than 2.4-kb and 2.1-kb RNAs (by the way, most such AREs are not in the “nontranslated region” of pgRNA. ELAVL1 is supposed to bind to AREs in the nontranslated region of RNA). Furthermore, pgRNA is unique in being encapsidated. Thus one concern is that such an experimental approach may be biased towards the identification of host factors interacting with encapsidated HBV RNAs or longer HBV RNA. Certainly subsequent tests seem to support a role of ELAVL1 in stabilizing total HBV RNAs as well, and the effect of ELAVL1 and CRM1 could be reproduced in core-minus mutant (Fig. 6). In some figures, reduction in HBsAg (mostly reflect 2.1-kb RNA) was striking. But according to Fig. 4L, ELAVL1 binding was 3.5kb RNA > 2.4kb RNA > 2.1-kb RNA, and absent in 0.7-kb RNA. Unfortunately, RNA quantification was PCR-based and can only be assigned to “pgRNA” (which should be both pgRNA and pcRNA due to larger size of pcRNA) and “total RNAs”. Thus the specific effect on 2.4-kb RNA, 2.1-kb RNA, and 0.7-kb RNA was unknown. By the way, in Fig. 4M, the 3’ end of the “preS1 mRNA” and “preS2/S mRNA” should be elongated to the same position as other RNAs.

*As this reviewer pointed out, HBV RNAs were hardly separated by qPCR assay. Thus, we transfected the plasmids expressing individual pcRNA, pgRNA, PreS1 mRNA, PreS2/S mRNA, and HBx mRNA into Huh7 cells* ***respectively****, as we specified in the figure legends of Fig 4.*

*We updated Fig 4M as the reviewer suggested.*

2. While conceptually the story told in this manuscript makes great sense, individual figures did show variability in quantitative values. Thus in Fig. 1D-F (based on HBV infection of HepG2/NTCP cells and transient transfection with ELAVL1 shRNAs), the effect of ELAVL1 silencing was most striking for intracellular HBV DNA, and least for extracellular HBeAg. The effects on extracellular HBV DNA, HBsAg and HBeAg were less striking on HepAD38 cell line (Fig. S2E) or on HepG2/NTCP cells with stable rather than transient ELAVL1 silencing (Fig. S2B). Although CMLD-2, an ELAVL1 inhibitor, dose-dependently reduced such HBV markers in HepG2/NTCP cells (Figs. 2C-E), the effect was modest (up to 40% reduction at the highest concentration).

*Thanks for pointing this issue out. The variations observed in the figures could be attributed to the different experimental conditions utilized. Factors such as the cell lines used (HepG2/NTCP, HepAD38), the method of ELAVL1 silencing (transient vs. stable), and the sensitivities and background of individual assays might contribute to the differences in observed outcomes. It's also important to consider that experimental error within the normal range can account for some variability in quantitative values across different assays.*

*For CMLD-2 treatment, we do not want to emphasize that CMLD-2 is a strong candidate for anti-HBV treatment. We only provide a proof of concept that targeting ELAVL1 maybe a potential way to inhibit HBV. We added a statement in the revised manuscript “Our study provides a proof of concept illustrating that targeting ELAVL1 holds promise as a potential approach to suppress HBV”.*

3. Only 3 earlier papers on PRE were cited (ref. 8, 29, 30). There should be much more work, such as J. Virol. 1994; 68: 3193-3199; and from another group: Nucleic Acids Res. 1998; 26: 4818-4827. According to the current manuscript, it appears that a role of PRE for HBV RNA export is no longer valid. I am unsure about that, especially considering that mutating all the 4 AREs in pgRNA only reduced its level by about 50%.

*These two papers were cited in our revised manuscript. Our study does not exclude the role of PRE in post-transcriptional regulation of HBV RNA. As we discussed, “It has been reported that the HBV PRE can enhance the expression of HBV transcripts and promote the nuclear export of unspliced HBV RNAs [14, 34-37]. In contrast, the PRE is not involved in the nuclear export of the full-length pgRNA that utilizes TAP/NXF1 dependent pathway [16]. In addition, there are evidences show that PRE contribute to pgRNA stability, but has little effect on its nuclear export [17]. Of note, these studies were mainly conducted in plasmid transfection models, and not validated in HBV infection models. Through a Genome-wide CRISPR Screen, Hyrina et al. uncovered ZCCHC14, together with TENT4A/B, interact with HBV PRE region and stabilize HBV mRNA by promoting 3′ end tailing, and thereby likely preventing degradation by exonucleases [38]. Here, we demonstrated that AREs are required for HBV RNA nuclear export via ELAVL1-CRM1 pathway. Together, these data suggesting different and distinct roles of PRE and ARE in HBV RNA post-transcriptional regulation.”*

Reviewer #2: minor issues:

1. repetition in line 515-566.

*Corrected.*

2. For RNAscope assay, what fluorescent microscope was used? The resolution seems low.

*The confocal laser scanning microscope we used to detect signals in cell was NIKON Eclipse Ti (software: Eclipse C2). The fluorescent channels we used were DAPI (EX 405 nm, EM 417-477 nm); probe dye 570 (EX 561 nm, EM 570-690 nm).*

*The confocal laser scanning microscope we used to detect signals in tissue was Leica TCS SP8 (software: LAS X Version 3.4.2.18368). The fluorescent channels we used were DAPI (EX 405 nm, EM 417-480 nm); probe dye 570 (EX 587 nm, EM 570-690 nm).*

*We have incorporated this information into the revised manuscript.*

*The resolution of the original images is 1024*1024. Presentative pictures are shown as blow.*

3.line 837, what is the dosage of AAV-1.2HBV?

*The dosage was 1 × 10^11^ vg. The information was provided in the section “Materials and methods”.*

Reviewer #3: (No Response)
