# Supplementary material for: Hepatitis B virus RNAs co-opt ELAVL1 for stabilization and CRM1-dependent nuclear export
Source: PLoS Pathog. 2024 Feb 2;20(2):e1011999. doi: 10.1371/journal.ppat.1011999 (PMC10866535; doi:10.1371/journal.ppat.1011999)
Supplement: Attachment — Submitted filename: PP-Response-2023-1-23.docx [file ppat.1011999.s014.docx]

Dear editor,

We would like to express our gratitude to the reviewers and editors for their input during this round of review. In this revised version, we conducted additional experiments involving evaluation of cell cycles in the presence of DMSO, and analysis of the effects of CMLD-2 on ELAVL1-HBV RNA binding by RNA binding protein immunoprecipitation assay (RIP). Please refer to point-by-point response provided below in *italic blue*.

**Part I - Summary**

Please use this section to discuss strengths/weaknesses of study, novelty/significance, general execution and scholarship.

Reviewer #1: The authors have responded to critiques raised in the previous round of review and added some additional data. I have no further comments.

Reviewer #2: In this revised manuscript, the authors addressed some of my comments by providing additional information and experimental evidence to consolidate the conclusion of the article. Nevertheless, I still have concern on the final proposed model on the mechanism of ELAVL1 on HBV RNA. Considering the sheer number of publications pointing to the pleotropic effects of this gene on cell biology (cell cycle, senescence, inflammation), rigorous examination of the other possible explanations should be taken into accoutn to minimize the self-fulfilled tendency of the manuscript.

Reviewer #3: The authors addressed most of the points that the reviewers raised. Incorporating all the methods in the manuscript and the figures in a clear way will be needed during the final stage but I don't have any other comments and based on the revision I recommend that the manuscript to be accepted.

Part II – Major Issues: Key Experiments Required for Acceptance

Please use this section to detail the key new experiments or modifications of existing experiments that should be absolutely required to validate study conclusions.

Generally, there should be no more than 3 such required experiments or major modifications for a "Major Revision" recommendation. If more than 3 experiments are necessary to validate the study conclusions, then you are encouraged to recommend "Reject".

Reviewer #1: none.

Reviewer #2: For example, although the authors used DMSO to stall the division of the cells, this does not translate to the complete absence of ELAVL1-mediated perturbation on the general cellular physiology. The author should at least mitigate this by providing RNA-seq data on the ELAVL1 knocked down cells in the absence or presence of HBV replication. The level of the perturbation of the general transcriptome on ELAVL1 silencing would generate an objective measure of the pleotropic effects of this gene.

*Thanks for pointing this issue out. Given the multifaceted roles of ELAVL1 in cellular physiology, it is important to note that its functions differ across various tissues and cell types, encompassing aspects such as RNA stability and nuclear export. Thus, it is not surprising that knockdown of ELAVL1 leads to disturbances many host genes. Consequently, it becomes challenging to identify specific genes or gene clusters that are involved in cell division and associated with the regulation of HBV replication in ELAVL1 knockdown cells by using RNA-seq.*

*To evaluate the impact of ELAVL1 knockdown on proliferation of the cells that we used in the presence of DMSO, we conducted flow cytometry analysis for cell cycle. The results demonstrate that the treatment of DMSO inhibits the progression of HepAD38 cells in the G1 phase of cell cycle, while ELAVL1 knockdown does not impact the cell cycle of HepAD38 cells treated with 2% DMSO.*

***HepAD38 proliferation was not affected by ELAVL1 knockdown in the presence of DMSO.*** *HepAD38 cells were treated with 2% DMSO for 2 days following ELAVL1 targeted siRNA transfection. Flow cytometry analysis was conducted utilizing the propidium iodide staining method to assess the levels of cells in distinct phases of the cell cycle.*

Also, there are a number of vulnerabilities in the presented data that are difficult to reconcile with the theory. For instance, the limited effects of CMLD-2 on the HBV transcripts and protein expression are an obvious counterargument for the proposed theory. Unfortunately, the authors chose to tune down this finding.

*Although CMLD-2 has been recognized as a potent inhibitor of ELAVL1-AREs binding in cancer cells, its biological effects and effective concentration vary among different cell lines (PMID: 36918912, 25750985, 31089242, 28855578). To investigate the compound's effects on ELAVL1-HBV RNA binding in our cell culture system, we conducted RNA immunoprecipitation (RIP) and observed that CMLD-2 led to only ~25% reduction in ELAVL1 antibody immunoprecipitation. The data suggested that the limited effects on HBV replication inhibition might be due to its weak suppression in ELAVL1-HBV RNA binding in hepatocyte carcinoma cells. The data were incorporated into new Fig 2.*

***CLMD-2 impaired ELAVL1-HBV RNA binding weakly.*** *HepAD38 cells were treated with 30 μM CMLD-2 for 2 days. RIP assay was performed to analysis ELAVL1-HBV RNA binding in the cells. Levels of HBV RNA in ELAVL1 antibody immunoprecipitation were detected by qPCR.*

Second, the S/E antigen level in the animal experiment (Fig. 3) showed a very late effect (3-4 weeks) on the ELAV1 knock down on the viral antigen. This seems to be at odds with the cell culture results, in which the effects on the viral RNA and antigen can be shown in days. Is there good explanation for this?

*Thank you for the question. Adeno-associated virus (AAV) exhibits the capability of sustaining long-term gene transcription with peak expression typically achieved at the third weeks of in vivo delivery, followed by sustained high-level expression for a duration exceeding five months. Consequently, researchers often evaluate the biological effects at or beyond the three-week time point (PMID: 28818946 (**1 month post AAV transduction); 30361367 (3* *weeks post AAV transduction); 30737013* *(4 weeks post AAV transduction)* *30361547(**4 weeks post AAV transduction); 23644515 (4 weeks post AA-shRNA transduction)). In line with this, we observed the* *impact on the ELAV1 knock down on the viral antigen since the 3rd week.*

Reviewer #3: no major issues

Part III – Minor Issues: Editorial and Data Presentation Modifications

Please use this section for editorial suggestions as well as relatively minor modifications of existing data that would enhance clarity.

Reviewer #1: none.

Reviewer #2: A minor issue.

The authors heavily used qRT-PCR to quantify the effects of ELAVL1 knockdown on different viral transcript. Some additional Northern blot in Figure 2 would be much more reassuring.

*We agree with the reviewer that additional Northern blot would be compelling. However, due to the limited source of P32 in China, the oversea ordering takes more than one month, so we could not complete the experiment in time. Although qRT-PCR, a widely used HBV RNA quantitative method, was intensively employed, it does not compromise the reliability of the data. In addition, for certain experiment (Fig 4L), individual HBV RNAs were studied.*

Reviewer #3: no minor issues but the figures in the revision had very low resolution.
